# Correction: Disentangling semantic and response learning effects in color-word contingency learning

**DOI:** 10.1371/journal.pone.0218222

**Published:** 2019-06-06

**Authors:** Sebastian Geukes, Dirk Vorberg, Pienie Zwitserlood

There are errors in Figs 1, 3, 4 and 8. In Fig 1, the numbers 3 and 4 are missing on the response box and a 5 is missing in “150 ms”. In Figs 3 and 4, the error bars in conditions ColPos and ColPosFix are distorted. In Fig 8, there is an error in the legend. The authors have provided corrected versions here.

**Fig 1:**
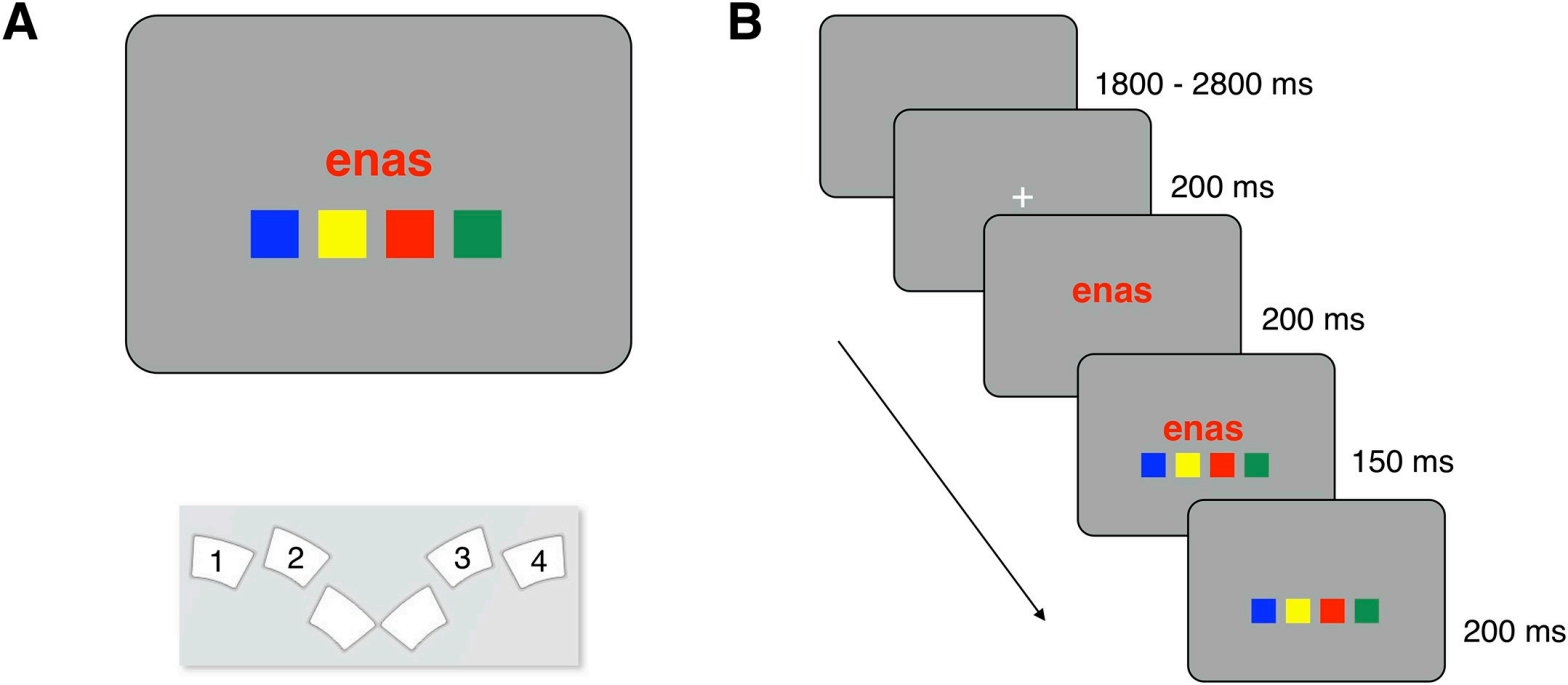
Stimulus display, spatial arrangement of response buttons, and trial timing. Panel A shows the typical stimulus display and response button layout in Experiments 1, 2, and 4. Panel B illustrates a typical trial. Numbers in Panel A schematically indicate which buttons of the response box were used in the experiments; actually, buttons were not labelled. The same timing of stimulus elements applied in all four experiments.

**Fig 3:**
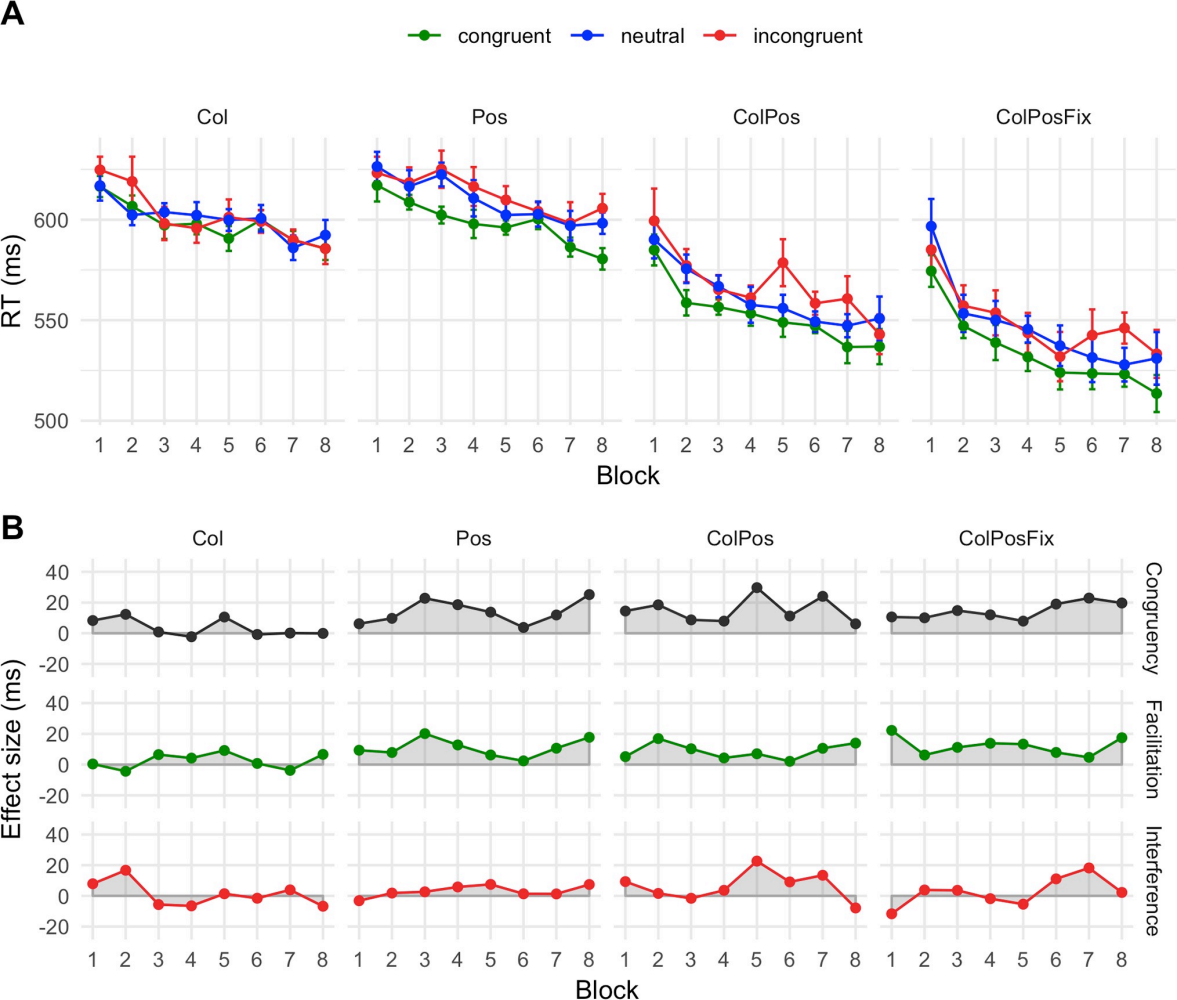
Mean response times (A) and net effects (B) in Experiment 1. Error bars are within-participant standard errors of the mean. Abbreviations: Col = color contingency, Pos = response position contingency, ColPos = both contingencies, ColPosFix = both contingencies plus fixed spatial arrangement of the response cues. Effects: Congruency = RTincongruent–_RTcongruent, Facilitation = RTneutral–_RTcongruent, Interference = RTincongruent–_RTneutral.

**Fig 4:**
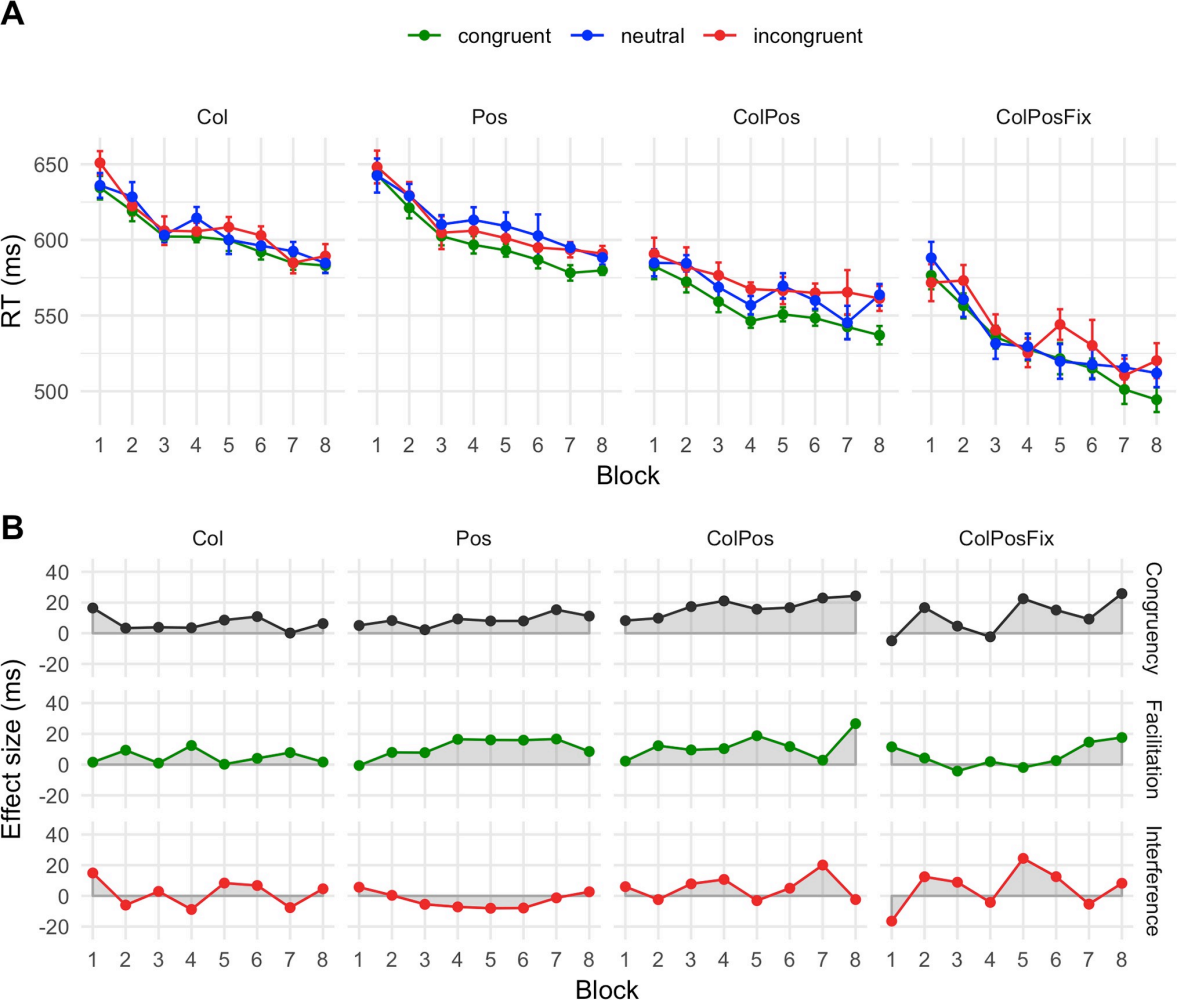
Mean response times (A) and net effects (B) in Experiment 2. Participants were informed about the contingency type before each upcoming block. Error bars and abbreviations as in Fig 3.

**Fig 8:**
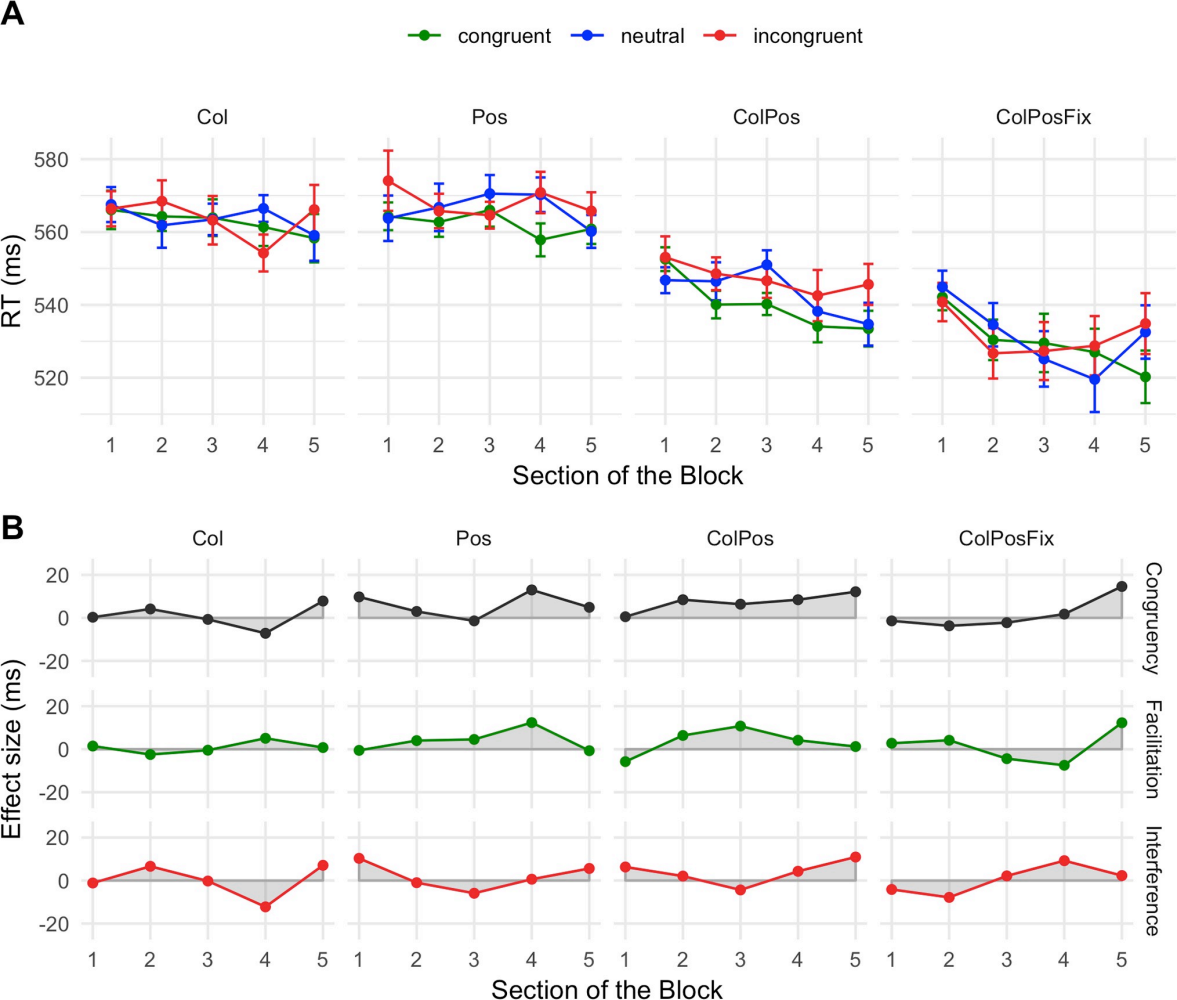
Mean response times (A) and net effects (B) in Experiment 4. Error bars and abbreviations as in Fig 3.
