# Supplementary material for: Disentangling semantic and response learning effects in color-word contingency learning
Source: PLoS One. 2019 May 6;14(5):e0212714. doi: 10.1371/journal.pone.0212714 (PMC6502354; doi:10.1371/journal.pone.0212714)
Supplement: S1 File — Error rates and matching performance for Experiments 1, 2, and 3. (PDF) [file pone.0212714.s001.pdf]

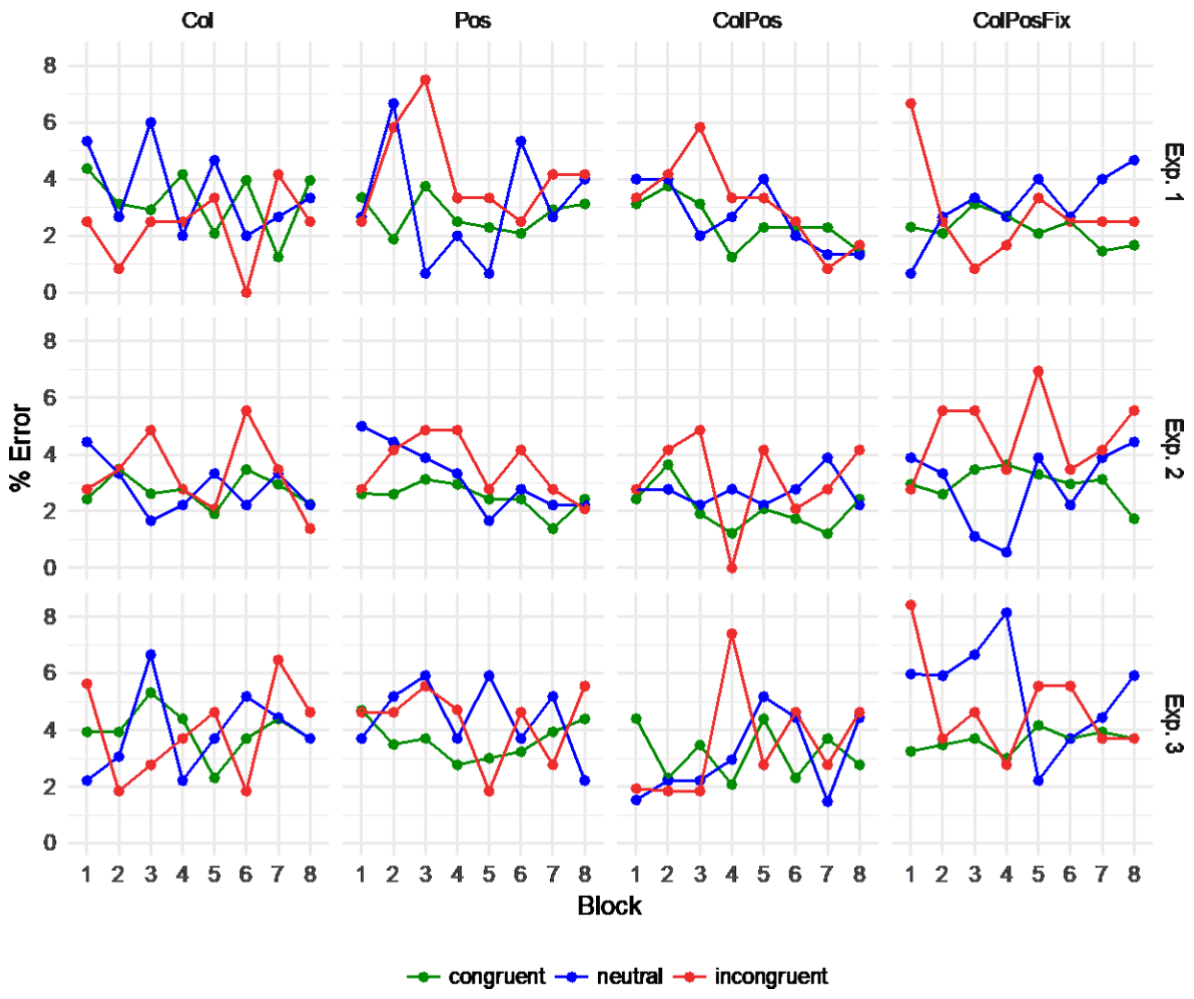

Figure A. Mean error rates in Experiments 1, 2, and 3.

Table A. Matching performance and Inferential statistics of in Experiment 1.

word type	*t(9)*	*p*	BF_{10}	BF_{01}
Matching by color				
Col	1.63	.068		1.19
ColPos	1.41	.096		1.49
ColPosF	2.86	.009	3.76	
Matching by position				
Pos	0.43	.338		2.99
ColPos	1.91	.045	1.15	
ColPosF	3.10	.007	5.11	

Note: t-tests are one-sample t-tests testing whether participants' matching of the respective four words to colors or positions was better than chance level (25%): Accordingly, one-sided p-values are reported. Scaled JZS Bayes Factors are also given, based on an r scale parameter of 0.707. BF_{10} denotes the Bayes Factor in favor of the alternative hypothesis (if $BF_{10} > 1$, evidence is in favor of above chance performance). When BF_{10} is below 1, that is, when evidence is in favor of the null hypothesis (chance performance or worse), the reciprocal BF_{01} (= 1/ BF_{10}) is given instead, to facilitate interpretation.

Table B. Matching performance and inferential statistics in Experiment 2.

word type	*M (SD)*	*t(11)*	*p*	*BF_{10}*	*BF_{01}*
Matching by color					
Col	.23 (.14)	0.80	.219		1.73
ColPos	.25 (.17)	1.00	.169		1.41
ColPosF	.42 (.26)	2.86	.008	8.23	
Matching by position					
Pos	.28 (.23)	1.24	.121		1.08
ColPos	.18 (.18)	-0.32	.623		4.32
ColPosF	.20 (.21)	0	.500		3.48

Note that neutral words were included in the matching task in Exp. 2 and chance level is thus at 20%.

Table C. Matching performance and inferential statistics in Experiment 3.

word type	*M (SD)*	*t(11)*	*p*	BF_{10}	BF_{01}
Matching by color					
Col	.31 (.30)	0.56	.297		1.98
ColPos	.33 (.35)	0.71	.250		1.72
ColPosF	.47 (.20)	3.41	.005	13.47	
Matching by position					
Pos	.25 (.19)	0	.500		2.97
ColPos	.41 (.30)	1.49	.090	1.35	
ColPosF	.16 (.19)	-1.43	.901		5.86

Note. As in Exp. 1, chance level is at 25%.

Table D. Summary statistics for all four experiments.

	COL			POS			COLPOS			COLPOSFIX		
EXP.	MEAN	S.D.	CE	MEAN	S.D.	CE	MEAN	S.D.	CE	MEAN	S.D.	CE
1	586	21	4	593	24	14	547	25	15	530	24	15
2	618	22	7	618	25	8	576	28	17	546	33	11
3	940	76	29	938	71	20	696	68	44	633	61	19
4	561	17	8	562	20	5	528	22	12	529	24	15

CE = net congruency effect RTincongruent - RTcongruent (Exp. 1-3: across all blocks; Exp. 4: last block segment, averaged across all blocks)
